# Temporal dynamics of categorization: forgetting as the basis of abstraction and generalization

**DOI:** 10.3389/fpsyg.2014.01021

**Published:** 2014-09-17

**Authors:** Haley A. Vlach, Charles W. Kalish

**Affiliations:** Department of Educational Psychology, University of Wisconsin–MadisonMadison, WI, USA

**Keywords:** spacing effect, forgetting, memory, category learning, categorization, novel noun generalization, generalization

## Abstract

Historically, models of categorization have focused on how learners track frequencies and co-occurrence information to abstract relevant category features for generalization. The current study takes a different approach by examining how the temporal dynamics of categorization affect abstraction and generalization. In the learning phase of the experiment, all relevant category features were presented an equal number of times across category exemplars. However, the relevant features were presented on one of two learning schedules: massed or interleaved. At a series of immediate and delayed tests, learners were asked to generalize to novel exemplars that contained massed features, interleaved features, or all novel features. The results of this experiment revealed that, at an immediate test, learners more readily generalized based upon features presented on a massed schedule. Conversely, at a delayed test, learners more readily generalized based upon features presented on an interleaved schedule, until information was no longer readily retrievable from memory. These findings suggest that forgetting and retrieval processes engendered by the temporal dynamics of learning are used as a basis of abstraction, implicating forgetting as a central mechanism of generalization.

## INTRODUCTION

Memory and categorization have historically been studied as separate components of cognition and operationally defined in a different manner. Memory has commonly been described as the process of encoding one item of information, storing that item of information, and then later retrieving that same item of information. Alternatively, categorization has commonly been described as the process of encoding multiple items of information, abstracting across items of information, storing an organized representation of that information, and then later retrieving knowledge in such a way that it can be generalized to new experiences. Although memory and categorization may involve different sub-processes, these two cognitive processes inevitably have common mechanisms. In recent years, researchers have recognized the need to outline these common mechanisms and have begun to examine relationships between memory and categorization (see Heit et al., 2012, for a review). We build upon this growing body of research by examining how temporal dynamics affect learners’ generalization.

### THEORIES/MODELS OF CATEGORIZATION AND GENERALIZATION

Generalization is central to categorization. After having seen many barking animals called “dog,” a learner will generalize that a novel animal, distinct from those past examples, is also called “dog.” This behavior depends on the process of abstraction. We define abstraction as the process of discounting some specific details of experience. In prototype theories of categorization, abstraction involves forming a stored summary representation of past examples that includes features common to category members, but leaves out (or minimizes) features that vary between category members. Alternatively, exemplar theories propose that abstraction occurs as a computation (e.g., similarity judgment) over representations of specific past examples (see Murphy, 2002, for a review). However instantiated, the nature of the abstraction determines generalization. How do we abstract information that is relevant and/or irrelevant for generalization?

**FIGURE 1:**
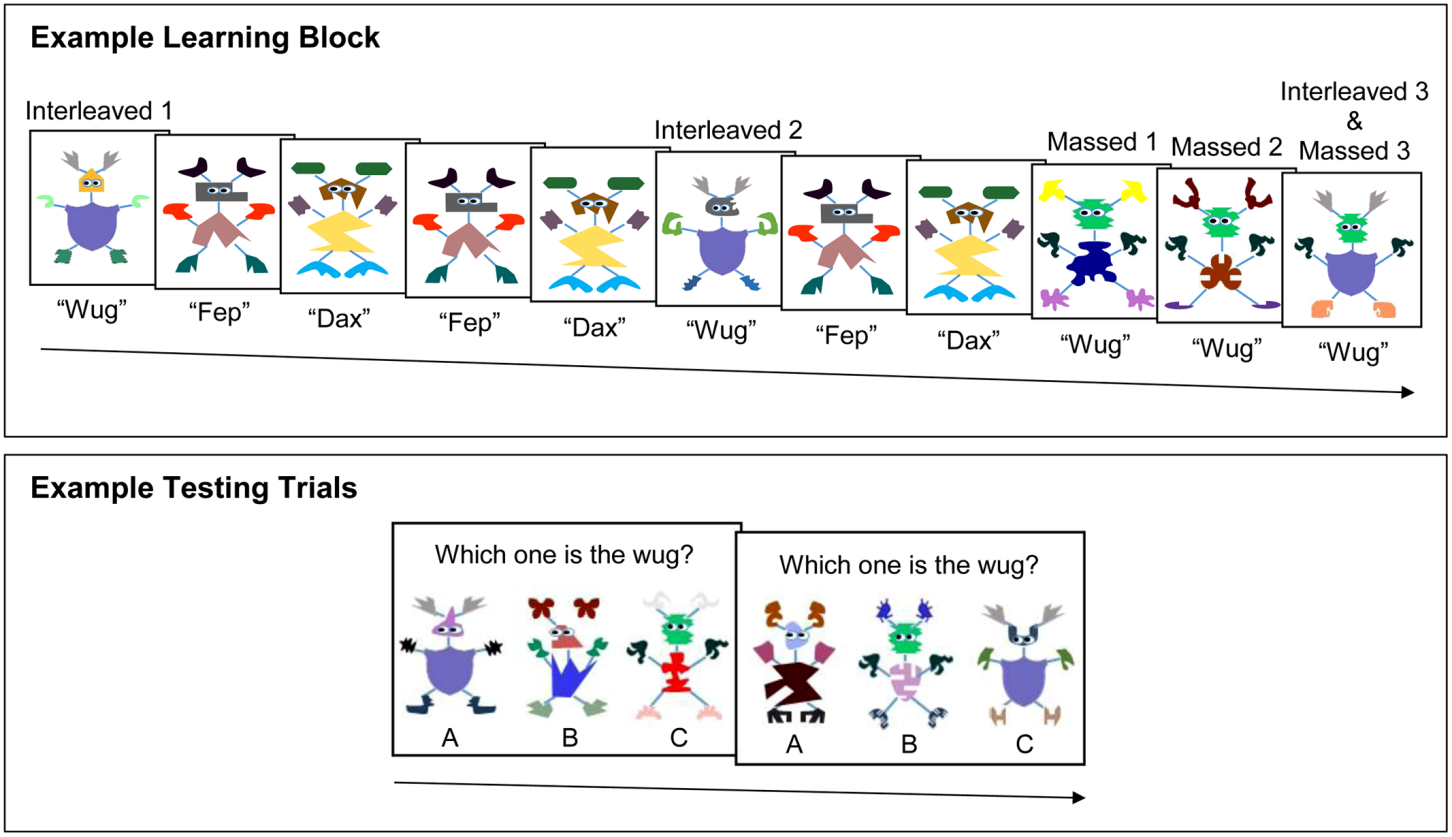
**Example of experimental procedure for one learning block (Learning Blocks 5 and 10, see **Table 1**) and corresponding testing trials.** In each learning block, two of the prototypical relevant features were presented in immediate succession (massed) and two were presented with four interleaved trials (interleaved). The massed and interleaved prototypical features were counterbalanced in each testing delay condition. At test, participants were asked to generalize to novel exemplars of the target category. One test choice consisted of massed prototypical features and novel features, one test choice consisted of interleaved prototypical features and novel features, and one test choice consisted of all novel features.

Theories and models of categorization have proposed that learners track associations between and co-occurrence of category features to abstract relevant information for generalization (e.g., Reed, 1972; Rosch and Mervis, 1975; Barsalou, 1985; Estes, 1986; Nosofsky, 1988; Rogers and McClelland, 2004 see Wills, 2013, for a review). That is, learners notice that some features occur frequently among category members, while other features occur less frequently. Those features reliably associated with category membership drive abstraction and generalization. For example, in a typical categorization paradigm, learners are presented with a series of novel objects or creatures (such as novel creatures used in this study, see **Figure 1** for examples). Each of these objects or creatures consists of features that can vary across instances of that category. Some of the features (e.g., a purple, round body) occur in many of the category exemplar presentations. Conversely, some features (e.g., pink, boxy feet) occur only in a few category exemplar presentations. At test, learners are asked to generalize what they have learned to novel category exemplars that include the low and/or high frequency features. This body of work has consistently found that learners are able to track the co-occurrence of features across category exemplars and that learners generalize based upon the frequency in which these features occur across instances of the category.

Given the theoretical framework of co-occurrence and association matrices as the basis for abstraction and generalization, research has focused on outlining how often information is presented affects learners’ categorization and generalization. However, the frequency at which information is presented to learners may not be the only basis on which learners abstract and later generalize information. In particular, the *timing* at which features are encoded and later retrieved for generalization may guide abstraction. Indeed, the temporal dynamics of learning may play an important role in the process of abstraction above and beyond that of the frequency at which information is presented. The current study examines this hypothesis.

### TEMPORAL DYNAMICS IN MEMORY AND GENERALIZATION

We ground our investigation of temporal dynamics and categorization in the memory literature, which has a long history of examining the temporal dynamics of learning. Since the late 1800s (Ebbinghaus, 1885/1964), research on human memory has demonstrated that distributing learning events across time promotes retention to a greater degree than massing learning events in immediate succession. This robust and highly replicable phenomenon is commonly called the *spacing effect* (for recent reviews, see Delaney et al., 2010; Toppino and Gerbier, 2014). Hundreds of articles in the memory literature, including several meta-analyses (e.g., Donovan and Radosevich, 1999; Cepeda et al., 2006), have observed a spacing effect across a wide variety of retention intervals. At an immediate memory test there is often higher performance for information presented on a massed learning schedule (e.g., Peterson et al., 1963; Glenberg, 1977). However, at a delayed test, participants consistently demonstrate higher performance for information presented on a spaced learning schedule compared to a massed learning schedule, until information is no longer retrievable from memory.

Why do we observe massing effects at an immediate test and spacing effects at a delayed test? Historically, there have been four classes of theories proposed to explain spacing effects: deficient processing theories (e.g., Hintzman, 1974), encoding variability theories (e.g., Glenberg, 1979), consolidation theories (e.g., Landauer, 1969), and study phase retrieval theories (e.g., Thios and D’Agostino, 1976). To date, the most predominant collection of theories are study-phase retrieval theories (or hybrid theories, such as study-phase retrieval theory and a component of another theory; see Delaney et al., 2010, for a discussion). Study-phase retrieval theories are predominant for several reasons. For example, the number of studies that have provided evidence for the study-phase retrieval account is larger than other theories, such as consolidation (Cepeda et al., 2006). Moreover, study-phase retrieval theories can account for many moderating and mediating factors of the spacing effect, such as why recognition may be required for a spacing benefit to emerge (e.g., Johnston and Uhl, 1976), why inhibited items show bigger spacing benefits than non-inhibited items (e.g., Anderson et al., 1994), and why difficult-to-learn material might result in shorter optimal lags (e.g., Paivio, 1974).

Indeed, study-phase retrieval theories have a parsimonious explanation for why we observe massing effects at an immediate test and spacing effects at a delayed test. According to study-phase retrieval theory, spaced schedules provide learners the opportunity to forget information during the intervals of time between presentations. Because forgetting occurs between learning events, learners have more difficulty retrieving prior learning events in spaced schedules compared to massed schedules. The enhanced difficulty that learners experience during spaced schedules engages them in more cognitive effort retrieving information, solidifying the memory trace and slowing the future forgetting rate of that information. Although the forgetting and retrieval difficulty experienced by learners in spaced schedules has benefits for long-term retention of information, there is often a detriment to immediate memory performance. Thus, at an immediate memory test, we observe massing effects because massed schedules prevent forgetting from occurring during learning, engendering easy immediate retrieval of information (and because of recency effects, see Peterson et al., 1963; Glenberg, 1977, for a discussion). However, after learning, the ability to retrieve massed information rapidly declines and information presented on a massed schedule is forgotten at a faster rate than a spaced schedule. Because massed information is forgotten at a faster rate than spaced information, there becomes a point in time where the two forgetting trajectories/curves cross over each other and spaced information becomes easier to retrieve than massed information. Consequently, we observe spacing effects at a delayed test, when learners can more readily retrieve information presented on a spaced schedule.

A recent trend in research on the spacing effect has been to examine spaced learning in tasks outside of the domain of memory, such as categorization and generalization tasks (Kornell and Bjork, 2008; Vlach et al., 2008, 2012, 2014; Kornell et al., 2010; Wahlheim et al., 2011; Kang and Pashler, 2012; Rohrer, 2012; Zulkiply et al., 2012; Birnbaum et al., 2013; McDaniel et al., 2013; Zulkiply and Burt, 2013; Carvalho and Goldstone, 2014). This body of work has revealed that spacing category exemplar presentations across time promotes categorization and generalization at a delayed test (e.g., Vlach et al., 2008, 2012; Birnbaum et al., 2013), as does interleaving non-category members between target category exemplar presentations (e.g., Kornell and Bjork, 2008; Wahlheim et al., 2011; Rohrer, 2012). In these studies, learners are presented with a series of novel objects (e.g., Vlach et al., 2008) or novel paintings (e.g., Kornell and Bjork, 2008) on a massed or spaced/interleaved schedule. All of the category exemplars presented during learning contain a central relevant feature (e.g., the same object shape or painter’s style) and, at test, learners are asked to generalize to novel exemplars with these features. Across studies, learners consistently have higher generalization performance for category exemplars presented on a spaced or interleaved schedule at a delayed test.

One theoretical account that has been proposed to explain the spacing effect in categorization is the forgetting-as-abstraction account (Vlach et al., 2008, 2012; for a review, see Vlach, 2014). The forgetting-as-abstraction account proposes that the intervals of time between category exemplar presentations allow learners the opportunity to forget information (as is also proposed in study-phase retrieval theory). The forgetting that occurs between learning events causes low frequency, irrelevant features to be forgotten at a faster rate than high frequency, relevant features. Relevant features are likely to be present at multiple learning events and thus reactivated in memory across category exemplar presentations. Spaced learning accelerates this process to a greater degree than a massed learning by engaging learners in more difficult retrieval of prior learning, in turn solidifying the memory trace and slowing down the forgetting rate of reactivated, relevant features (in line with dynamics proposed by study-phase retrieval theory). Alternatively, low frequency, irrelevant features are not likely to be present at multiple learning events and thus not reactivated in memory. As a result, relevant information is more readily retrievable than irrelevant information, which supports generalization at test. In sum, the forgetting and retrieval dynamics occurring during spaced learning speeds up the abstraction of high frequency, relevant features by making them more readily retrievable at later points in time.

One assumption of the forgetting-as-abstraction account is that learners rely on the ease at which they retrieve information as an indicator of which category features are more relevant than other features for generalization. However, this assumption has yet to be empirically tested in the context of spaced/interleaved learning and categorization studies. If it is the case that learners use the retrievability of information as a guide for generalization, this would provide additional support for the forgetting-as-abstraction account. Moreover, it would also expand the forgetting-as-abstraction account by suggesting that general patterns of forgetting alone could serve as a form of abstraction. That is, the forgetting trajectories/curves of information presented on massed and spaced/interleaved learning could operate as a form of abstraction above and beyond other forms of abstraction, such as tracking the frequency of category features. Indeed, according to study-phase retrieval theory, massed and spaced/interleaved learning have different underlying forgetting trajectories/curves which make information more readily retrievable at different points in time (i.e., immediate vs. delayed tests).

To date, studies on categorization have traditionally manipulated the frequency of category features, which learners can track and use to abstract relevant and irrelevant features for generalization. To test the prediction that forgetting and retrievability of information alone can be the basis of abstraction, the current study held constant the frequency with which features were associated with a category. Instead of manipulating the frequency of category features, the current study manipulated the presentation timing of relevant category features. Thus, the current study examined the question: Can the temporal dynamics of learning affect the degree to which equally associated features are used as the basis of abstraction and generalization?

### CURRENT STUDY

This study manipulated the timing at which features were presented during category learning. For each category, one set of features was presented on a massed schedule and one set of features was presented on an interleaved schedule. Although the presentation timing of the feature sets varied (i.e., massed vs. interleaved schedule), all of the features were associated with the category an equal number of times during learning. At a forced-choice generalization test, learners were presented with one novel exemplar that contained massed features of the category, one novel exemplar that contained interleaved features of the category, and a distractor object that contained all novel features. If learners use solely the co-occurrence or frequency of features for abstraction, we expected to observe no differences at test in choosing the novel exemplar with massed features vs. the novel exemplar with interleaved features.

However, we predicted that the presentation timing and temporal dynamics of category learning and generalization would affect the manner in which learners abstract information that is relevant for generalization. In particular, we hypothesized that learners use the retrievability of learned information to guide their abstraction of relevant information for generalization. Based upon the spacing effect literature and study-phase retrieval theory, we expected that features presented on a massed schedule would be more retrievable than features presented on an interleaved schedule immediately after learning. Thus, we predicted that participants would choose the novel exemplar with massed features more often than the novel exemplar with interleaved features at an immediate test. Conversely, we expected that features presented on an interleaved schedule would be more retrievable than features presented on a massed schedule after a delay. Consequently, we predicted that participants would choose the novel exemplar with interleaved features more often than the novel exemplar with massed features at a delayed test. Finally, if information is no longer readily retrievable in memory, we expected to observe chance performance across the three test choices.

To examine these possibilities, learners’ generalization was tested at two immediate tests (immediately after each learning block and immediately after all learning blocks) and two delayed tests (at a 3 min delay and at a 5 min delay). These delays were chosen to test our hypotheses regarding temporal dynamics, as outlined above, and parallel testing delays commonly used in studies of spaced learning and categorization (e.g., Kornell and Bjork, 2008; Vlach et al., 2008). In sum, these learning and testing conditions provided a direct examination of how the temporal dynamics of categorization affect the manner in which learner’s abstract relevant information for generalization.

## MATERIALS AND METHODS

### PARTICIPANTS

The participants were 149 undergraduate students recruited from the undergraduate subject pool of the Department of Educational Psychology at the University of Wisconsin–Madison. Participants received course credit for their participation. Participants were assigned to one of the four testing delay conditions, resulting in 37 participants tested immediately after each learning block, 36 participants tested immediately after all learning blocks, 35 participants tested with a 3 min delay, and 41 participants tested with a 5 min delay.

### DESIGN

The study was a 2 (Feature Presentation Timing) × 4 (Testing Delay) design. Feature Presentation Timing (massed or interleaved) was a within-subjects factor and Testing Delay (immediately after each learning block, immediately after all learning blocks, after a 3 min delay, or after a 5 min delay) was a between-subjects factor.

### APPARATUS AND STIMULI

Participants saw a series of novel objects and novel linguistic labels (see **Figure 1**, for examples). Novel objects were presented on a 13 inch laptop screen and the novel linguistic labels were presented through the computer’s speakers.

The novel objects consisted of five features: a head, body, ears, hands, and feet. Each feature varied in color and shape (e.g., square red feet or round blue feet). Four of the features were chosen to be the relevant features of each target category and one feature was chosen to be an irrelevant feature of the category. As can be seen in **Table 1**, the assignment of the four relevant features was counterbalanced across the target categories/learning blocks so that each feature was a relevant feature the same number of times across the entire experiment.

**Table 1:** Distribution of relevant and irrelevant features of target categories by learning block.

	Head	Body	Ears	Hands	Feet
Learning Block 1 and 6	0	1	1	1	1
Learning Block 2 and 7	1	0	1	1	1
Learning Block 3 and 8	1	1	0	1	1
Learning Block 4 and 9	1	1	1	0	1
Learning Block 5 and 10	1	1	1	1	0

*A “0” indicates that a feature was an irrelevant feature of the target category. A “1” indicates that a feature was a relevant feature of the target category. The distribution outlined in this table demonstrates the each feature (head, body, ears, hands, or feet) was assigned to be an irrelevant or relevant feature of the target category an equal number of times during the experiment.*

*The novel linguistic labels for each object and target category were randomly assigned. All linguistic labels followed the phonotactic probabilities of English (e.g., “wug,” “dax,” “fep,” etc). Each of the linguistic labels was used in only one learning block of the experiment. As a result, there were a total of 30 novel labels used across the entire experiment.*

### PROCEDURE

The study began with the experimenter reading instructions to the participants. The instructions were to view a series of objects that they had never seen before. After the experimenter finished reading the instructions to the participants, the first learning block would begin.

The experiment consisted of ten learning blocks, each with two corresponding testing trials. Overall, the experiment lasted about 9 min; the experiment lasted longer if participants were in one of the delayed testing conditions (e.g., 5 min delayed testing condition added 5 min to the experiment).

#### Learning blocks

Each learning block presented participants with one target category. The learning blocks consisted of 11 learning trials, five of which were target category presentations (e.g., the “wugs,” in **Figure 1**, top panel) and six of which were filler trials with other novel objects (e.g., the “feps” and “daxes,” in **Figure 1**, top panel). The duration of each trial was 4 s, for a total duration of 44 s per learning block.

In each learning block, two of the prototypical relevant features were presented on a massed schedule and two of the prototypical relevant features were presented on an interleaved schedule. Assignment of features to massed and interleaved schedules was counter-balanced across learning blocks and participants. Learning blocks followed a consistent order of presentation. Trials 1 and 6 always presented target category members (e.g., “wugs”) with two features which were the same color and shape in both trials (e.g., the body and ears in **Figure 1**). These two consistent features were the prototypical relevant features assigned to the interleaved schedule. Trials 9 and 10 always presented target category members (e.g., “wugs”) with two features which were the same color and shape in both trials (e.g., the head and hands in **Figure 1**). These two consistent features were the prototypical relevant features assigned to the massed schedule. Trial 11 always presented a category member with both the massed and interleaved prototypical relevant features. Thus, Trial 11 represents the prototypical category member. The category members in Trials 1 and 6 shared interleaved features with the prototype, while the category members in Trials 9 and 10 shared massed features with the prototype. All category members received the same linguistic label (e.g., “wug” in **Figure 1**).

Trials 2, 3, 4, 5, 7, and 8 were filler trials. Filler trials consisted of one of two non-category members (e.g., “fep” and “dax” in **Figure 1**). Non-category members differed on all five features from the target category members. Each non-category member appeared three times in a learning block and was the same object in each trial. Thus, there was no contrast category in each learning block (as sometimes included in other paradigms, see Kornell and Bjork, 2008; Carvalho and Goldstone, 2014); participants were only asked to learn and categorize one target category per learning block.

This learning block design had three critical properties. First, there was an equal co-occurrence of each prototypical relevant feature of the target category. Across trials in the block, participants saw three target category members with interleaved prototypical features (1, 6, and 11) and three target category members with massed prototypical features (9, 10, and 11). Second, there was an equal number of intervening trials between each of the interleaved feature presentations; there were four intervening trials between each interleaved feature presentation. Third, because every prototypical feature was presented on the last learning trial, there was an equal duration between the last exposure to a particular feature and the testing trials in which learners generalize these features to novel category exemplars. In sum, the only dimension on which the feature presentation differed was the presentation timing of the relevant prototypical features: half of the prototypical features were presented on a massed schedule and half were presented on an interleaved schedule.

#### Test trials

Participants were presented with two forced-choice generalization trials for each learning block. This resulted in 20 testing trials during the experiment. In the immediately after each block testing condition, the two test trials were presented after Trial 11 of each learning block. In the immediately after all learning blocks testing condition, the testing trials were presented after all ten learning blocks (i.e., after Trial 110). Finally, in the 3 and 5 min delay testing conditions, the testing trials were presented 3 or 5 min after the last learning trial (i.e., 3 or 5 min after Trial 110). Participants in the delay conditions played the game Angry Birds^©^ during the delay period. Testing trials always occurred in an order consistent with the learning blocks (e.g., the first testing trial corresponded to the first learning block).

In test trials, depicted in **Figure 1**, bottom panel, participants saw three novel objects. The task was to pick an object that corresponded with the category label presented during the corresponding learning block (“Which one is the wug?”). One object consisted of the two prototypical interleaved features of the target category (e.g., the body and ears of a “wug”) and three novel features. A second object consisted of the two prototypical massed features of the category (e.g., the head and hands of a “wug”) and three novel features. A third object was an unfamiliar novel object; this object was not presented during any of the learning blocks and all features were novel. After choosing an object (labeled A, B, or C, see **Figure 1**) participants would proceed to the next test trial until all test trials were complete.

## RESULTS

A central goal of this experiment was to examine whether the presentation timing of different features during learning (i.e., massed or interleaved schedule) would affect generalization performance. If memory processes are critical to categorization and generalization, we expected to observe differences across delay conditions in the features that participants use to generalize to novel category members. In contrast, if memory processes are not critical to categorization and generalization beyond retrieving co-occurrence information, we expected to observe no differences in which features are used to generalize because all of the prototypical relevant features were presented an equal number of times during learning. Beyond a general effect of delay (i.e., diminishing performance over time), we tested specific predictions about how generalization performance may change across time. Based upon the hypotheses, participants should have been more likely to generalize using massed features than interleaved features at the immediate tests. Furthermore, participants also should have been more likely to use interleaved features than massed features at a delayed test, unless participants were no longer able to generalize what they had learned.

**FIGURE 2:**
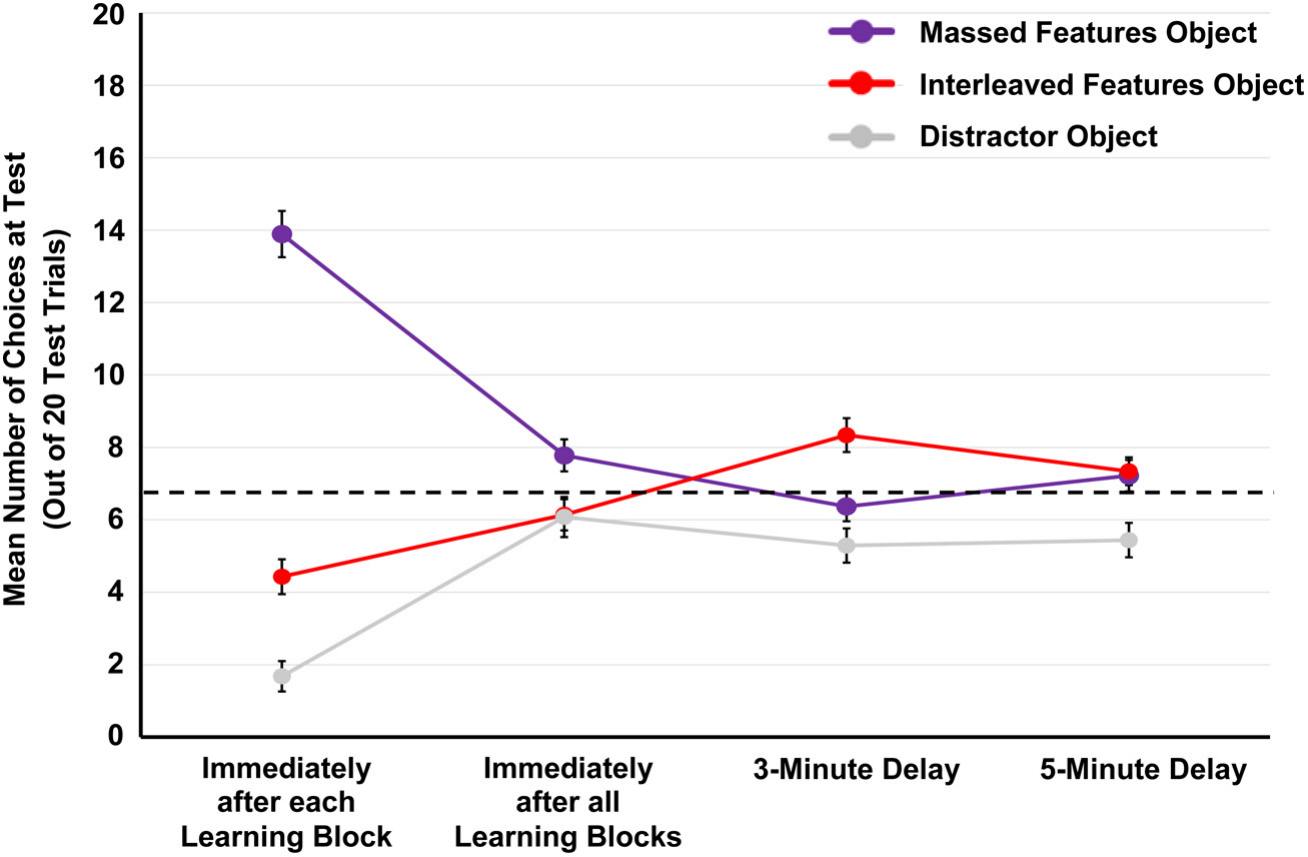
**Mean number of times participants selected the massed features object, interleaved features object, and distractor object across 20 generalization test trials by testing delay (immediately after each learning block, immediately after all learning blocks, at a 3 min delay, and at a 5 min delay).** Errors bars represent one SE. Dotted lined represents chance performance.

As can be seen in **Figure 2**, participants appeared to demonstrate differences in generalization performance between the massed and interleaved items across the testing delay conditions. To examine this possibility, a difference score was calculated between the massed and interleaved test choices at each testing delay. Next, a univariate ANOVA was conducted with difference score (massed minus spaced) as the outcome variable and testing delay (immediately after each learning block, immediately after all learning blocks, 3-min delay, or 5-min delay) as a between-subjects factor. The analysis revealed a main effect of testing delay, *F*(3,145) = 39.410, *p* < 0.001, = 0.449. Thus, there were significant differences in the number of massed vs. interleaved test choices across the testing delays.

To examine the nature of the differences across testing delays, each difference score was compared to 0 (no difference) using a one-sample *t*-test. In the immediately after each learning block condition, the difference score (*M* = 9.46, SD = 6.384) was significantly above 0, *t*(36) = 9.013, *p* < 0.001. Likewise, in the immediately after all learning blocks condition, the difference score (*M* = 1.64, SD = 4.051) was significantly above 0, *t*(35) = 2.428, *p* = 0.020. Thus, in the two immediate testing delay conditions, participants generalized based upon massed features to a greater degree than interleaved features. In the 3 min delay condition, the difference score (*M* = -1.97, SD = 4.389) was significantly below 0, *t*(34) = -2.657, *p* = 0.012. Given the negative difference score, this indicates that participants generalized based upon interleaved features to a greater degree than massed features. Finally, at the 5 min delay, the difference score (*M* = -0.12, SD = 4.261) was not significantly different than 0, *t*(40) = -0.183, *p* = 0.856.

Why was there no difference between massed and interleaved test choices at the 5 min delayed test? Upon visually inspecting **Figure 2**, it appeared that participants were choosing both the massed and interleaved test choices at chance performance (1/3 of the 20 test items). To examine this possibility, we compared the two test choices to chance performance; these results revealed that there was no significant difference from chance performance for the massed features test choice, *t*(40) = 1.278, *p* = 0.209, and a marginally significant difference from chance for the interleaved features test choice, *t*(40) = 1.760, *p* = 0.086. However, participants chose the distractor object significantly less than chance, *t*(40) = 2.584, *p* = 0.014. These findings suggest that, by the 5 min delay, participants may have mostly forgotten both the massed and interleaved features, and thus they were not generalizing based upon memory for features, but randomly guessing between the massed and interleaved items (and perhaps distractor items) at the test.

In sum, the results of this experiment revealed that the timing at which category features were encoded and then later generalized had a significant impact on generalization performance. In the two immediate testing conditions, we observed that participants chose the massed features object significantly more than the interleaved features object. However, at the 3-min testing delay, we observed that participants chose the interleaved features object significantly more than the massed features object. Finally, at the 5-min testing delay, we observed chance performance.

Why do we observe differences in generalization across time? Taken together, these findings suggest that learners are abstracting and generalizing knowledge *based upon what they remember*, rather than generalizing solely on the basis of a category prototype or set of co-occurrence statistics across exemplars. As a result, understanding the nature of categorization and generalization likely requires understanding how memory and categorization processes interact over timescales, as outlined in the section “Discussion.”

## DISCUSSION

This study manipulated the timing at which relevant category features were presented during category learning. Although the presentation timing of the features varied, all of the relevant features associated with category membership were presented an equal number of times during learning. Based upon traditional models of categorization, which are largely grounded in frequency and co-occurrence statistics as the basis for abstraction, we should have observed no differences in performance across the generalization tests. Instead, we observed that participants had different patterns of performance based upon the timing at which they encoded category features (i.e., massed or interleaved schedule) and were asked to generalize (i.e., immediate or delayed test). These results have implications for extant theories of categorization and highlight the importance of looking to domains beyond categorization, such as memory, for elucidating the mechanisms that influence abstraction and generalization.

### HOW DO THE TEMPORAL DYNAMICS OF LEARNING AFFECT ABSTRACTION AND GENERALIZATION?

Theoretical accounts of how the temporal dynamics of learning affect memory have a long history in psychological science (dating back to Ebbinghaus, 1885/1964). As mentioned in the Introduction section, historically there have been four classes of theories to explain spacing effects in memory, with study-phase retrieval theory being the most parsimonious and predominate to date. Only recently have spacing effects been tested in the context of categorization and generalization tasks (e.g., Kornell and Bjork, 2008; Vlach et al., 2008), and thus there are only two accounts that have been proposed to explain why spaced/interleaved learning promotes generalization. The current results expand upon these recent theoretical accounts.

One theoretical account is the discrimination account, which argues that interleaved learning supports generalization by facilitating the discrimination of features that are relevant for generalization within and/or between categories (Kurtz and Hovland, 1956; Kang and Pashler, 2012; Rohrer, 2012; Zulkiply and Burt, 2013; Carvalho and Goldstone, 2014). For example, in these studies (e.g., Zulkiply and Burt, 2013; Carvalho and Goldstone, 2014), discriminability within and between categories is manipulated by changing number of times (i.e., frequency) that relevant and/or irrelevant category features co-occurred at each category exemplar presentation. These studies have demonstrated that spaced/interleaved schedules promote generalization performance for low-discriminability categories, but perhaps not high-discriminability categories. Consequently, it has been proposed that spaced/interleaved learning promotes generalization by supporting certain types of discriminations within and between categories.

In the current experiment, the co-occurrence and similarity of each feature was equivalent within the target categories (i.e., equally weighted/presented relevant features). There was also only one target category per learning block, with no contrast category, which is often included in studies examining discrimination processes (e.g., Zulkiply and Burt, 2013; Carvalho and Goldstone, 2014). Thus, it is unclear how discrimination or interference (such as retroactive interference between features/categories) would have affected generalization in this task. Moreover, it is unclear how discrimination or interference would produce a massing effect at an immediate test and an interleaving effect at a delayed test. In sum, the current study does not provide direct evidence against the discrimination account or potential interference effects because the experimental paradigm was not designed to examine or test for these dynamics. However, this work does suggest that discrimination and/or interference are not the only mechanisms underlying the temporal dynamics of learning in generalization (also see Birnbaum et al., 2013, for converging evidence) and that other processes may engender massing and spacing/interleaving effects in categorization tasks.

The current results provide further evidence supporting another recent theoretical account, the forgetting-as-abstraction account (Vlach et al., 2008, 2012; see Vlach, 2014, for a review). As outlined in the section “Introduction”, this account proposes that forgetting can promote abstraction of high frequency features and, consequently, promote generalization. The current study extends this theoretical account by demonstrating that forgetting alone can produce abstraction and generalization by making some aspects of past experience more available than others. In particular, massed features will be more available at an immediate test. Conversely, spaced/interleaved features will be more available at a delayed test. This differential in retrievability will affect generalization; the more readily available features will be more likely to be used as the basis of abstraction and influence classification of new instances.

As an illustration of the forgetting-as-abstraction account, consider a participant’s challenge of determining which test item is the fep. To make this judgment, the participant must recall something of the feps (and perhaps non-feps) encountered in the past. In an exemplar model (e.g., Nosofsky, 1988), these will be memories of items. The current study suggests that items presented on an interleaved schedule will be more available during categorization than those presented on a massed schedule at a delayed test. That is, when the participant recalls the feps she has seen, she is more likely to bring to mind interleaved items than massed items. To make a categorization judgment, the participant compares the to-be-judged item(s) to the set of items recalled from memory. If the set of recalled items is biased toward interleaved items, then features that co-occurred with the category label in those items will influence the similarity computation more so than co-occurrences in the massed items. A participant might remember many feps with blue hands (interleaved items) but few with square bodies (massed items), for example. Because only some of the information about the category is recalled and used for categorization the participant has effectively constructed an abstraction. The participant will be highly sensitive to variation on some features (e.g., hands) and relatively insensitive to variation on others (e.g., bodies). The representation of fep is abstract because only some features matter. Blue hands will be important in identifying new feps but the participant will accept a range of body-types^1^. This kind of abstraction through weighting of a similarity computation is a central feature of exemplar models (e.g., Nosofsky, 1988).

In any account of categorization, some features must be weighted more heavily than others. For example, the sound an animal makes has a greater influence on its classification as a dog or cat than does its color. Sound is weighted more heavily than color. A large body of literature on categorization demonstrates that these feature weights are affected by patterns of frequency and co-occurrence (see Murphy, 2002, for a review). The current study suggests a second set of influences on feature weights: the temporal dynamics of learning, and the underlying forgetting and retrieval dynamics engendered by these dynamics. Indeed, future research should build upon this work by examining the relative influences of frequencies and forgetting, as outlined below.

## CONCLUSION AND FUTURE DIRECTIONS

The current study highlights that there are several ways in which forgetting influences abstraction, categorization, and generalization. Consequently, this brings to question how forgetting might interact with frequency and co-occurrence information across timescales. For example, would differential rates of forgetting lead people to sometimes classify based on less frequently associated features? Would reliable associations between features change rates of forgetting? A second set of future questions concern the psychological mechanisms of frequency weighting and forgetting. Learners may find it easier to remember a dog’s color than its bark, but base classification and generalization decisions on barking. Conversely, the effects of forgetting may be interpreted or experienced in terms of frequencies. Would participants in a task like the one reported above recognize that the feature frequencies were equal, or would forgetting lead to something like an illusory correlation?

On a final note, it is worth pointing out that the close relationship between memory and categorization implied by forgetting-as-abstraction account has implications for understanding memory as well. In particular, the way that information is to be used for categorization may affect the way information is later remembered and forgotten. Several recent studies have started to outline the cases in which categorization affect memory in this manner. For example, people remember more detail about stimuli in the context of a memory task than in the context of a categorization task (Sloutsky and Fisher, 2004). Similarly, young children recall more unique features of individuals when they think they are learning something specific to that individual than when they think they are learning something general about a class. However, they show better memory for properties the people displayed when learning about a class rather than an individual (Riggs et al., 2014a,b). We expect that these kind of reciprocal effects are the rule rather than exception. Indeed, memory and categorization are intimately linked and future work should continue bridge these historically disparate fields of work.

## Conflict of Interest Statement

The authors declare that the research was conducted in the absence of any commercial or financial relationships that could be construed as a potential conflict of interest.

## Footnotes

1 Prototype models have a similar account of abstraction, but it occurs as certain features get strengthened or weakened in the prototype representation (see Posner and Keele, 1970). Interleaved features may take on greater weight in the prototype and drive comparison to test items more so than massed features.
